# A field evaluation of two vaccines against *Mycoplasma hyopneumoniae* infection in pigs

**DOI:** 10.1186/1751-0147-56-24

**Published:** 2014-04-16

**Authors:** Charlotte S Kristensen, Jens Vinther, Birgitta Svensmark, Poul Bækbo

**Affiliations:** 1 Danish Agriculture and Food Council, Pig Research Centre, Vinkelvej 11, DK-8620 Kjellerup, Denmark

**Keywords:** *Mycoplasma hyopneumoniae*, Enzootic pneumonia, Vaccination, Productivity

## Abstract

**Background:**

A field trial was carried out with two *Mycoplasma hyopneumoniae* vaccines in order to investigate the benefit of vaccination under field conditions in modern Danish pig production facilities with pigs being positive for *M. hyopneumoniae*. The *M. hyopneumoniae* infection of the herd was confirmed through blood samples that were positive for antibodies against *M. hyopneumoniae* combined with gross lesions of the lungs related to *M. hyopneumoniae* at slaughter and detection of *M. hyopneumoniae* by polymerace chain reaction in these lesions.

**Results:**

A total of 2,256 pigs from two herds were randomly divided into three groups. Group 1 received 2 mL ThoroVAX^®^VET, Group 2 received 1 mL Ingelvac^®^MycoFLEX, and Group 3 was a non-vaccinated control group. The vaccination was performed by a person who was not involved in the rest of the trial and vaccination status thereby blinded to the evaluators.

The prevalence of lung lesions related to *M. hyopneumoniae* were significantly lower for pigs vaccinated with ThoroVAX^®^VET but not for pigs vaccinated with Ingelvac^®^MycoFLEX^®^, when compared to non-vaccinated pigs. There was no significant effect of vaccination on growth rate, antibiotic consumption or mortality.

**Conclusion:**

This trial demonstrated that vaccination with Thoro^®^VAX VET was effective in reducing the prevalence of lung lesion in pig units infected with *M. hyopneumoniae*.

## Background

Swine enzootic pneumonia causes major economic losses in the pig industry worldwide [1]. *Mycoplasma hyopneumoniae* is the primary agent responsible, although secondary infections can increase the severity of the disease [2,3]. This interaction with both bacterial and viral pathogens is referred to as porcine respiratory disease complex [4]. Therefore, control of *M. hyopneumoniae* is critical in reducing economic losses in the pig industry.

Active immunisation of pigs has been recommended for preventing *M. hyopneumoniae* disease and reduced productivity. A meta-analysis of the effect of *M. hyopneumoniae* vaccines showed an increase in the average daily gain (ADG) of 20 g for vaccinated finishers compared with non-vaccinated pigs [5].

Pig production levels in Denmark are very high; the ADG from a body weight of 30–100 kg is 905 g and the mortality is 3.6% [6]. This high level of productivity is mainly due to modern production units operated on an all-in/all-out basis and with a high level of biosecurity. Due to the widespread occurrence of *M. hyopneumoniae*, a large number of pigs are vaccinated against *M. hyopneumoniae*. Annually, more than 11 million doses of vaccine against *M. hyopneumoniae* are sold in Denmark for the production of 30 million pigs, of which nine million nursery pigs are exported, and 21 million pigs are slaughtered in Denmark.

It could be questioned whether the benefit of vaccination is still valid in modern pig production systems with a high level of biosecurity, which are becoming more and more widely adopted worldwide. Therefore, the aim of this study was to evaluate the effect of two vaccines against *M. hyopneumoniae* on the prevalence of lung lesions, ADG, antibiotic consumption and mortality in such facilities.

## Methods

### Selection of herds

Two herds were selected for this trial on the basis of a history of clinical respiratory problems.

Herd A was located on a small island close to Funen. It was a specific pathogen free (SPF) herd but infected with *M. hyopneumoniae*, Porcine reproductive and respiratory syndrome virus (PRRS) types 1 and 2 and housing only nursery and grower/finisher pigs. The herd received 575 four-week-old piglets every 4th weeks, which were assigned to two sections. The two sections of nursery pigs were commingled in one grower/finisher section. Both nursery and grower/finisher units were operated on an all-in/all-out basis by section. The pigs were treated for oedema disease with antibiotics during the first three weeks after arrival. Before inclusion in the trial, lung lesion scores were evaluated in 30 pigs from one delivery batch as described by [2]. This evaluation found that 87% of the pigs had catarrhal bronchopneumonia. *M. hyopneumoniae* was detected by an in-house polymerase chain reaction (PCR) assay at the Danish Veterinary Institute (Copenhagen, Denmark) from one lung with catarrhal bronchopneumonia, and antibodies against *M. hyopneumoniae* were detected by enzyme-linked immunosorbent assay (ELISA) at the Danish Veterinary Institute in ten out of ten finishers [7].

Herd B was located in the middle of Jutland. It was a SPF herd infected with *M. hyopneumoniae* and *Actinobacillus pleuropneumonia* serotypes 6 and 12. The herd was a farrow-to-finish production unit consisting of 380 sows producing between 300 and 500 pigs every second week. At weaning, at four weeks of age, the pigs were moved to a section that was operated on an all-in/all-out basis. The grower/finisher units were mainly operated on an all-in/all-out basis by section. Before inclusion in the trial, lung lesion scores were evaluated in 30 pigs from one delivery batch as described by [2]. This evaluation found that 60% of the pigs had catarrhal bronchopneumonia. *M. hyopneumoniae* and antibodies were detected as for herd 1.

Before inclusion the herd owners had to sign informed client consent.

### Vaccines

The vaccines tested in this study were marketed as ThoroVAX^®^VET (M + PAC outside Denmark; MSD Animal Health, Ballerup, Denmark) and Ingelvac^®^MycoFLEX (Boheringer Ingelheim Animal Health, Copenhagen, Denmark). Both vaccines contain inactivated *M. hyopneumoniae*. ThoroVAX^®^VET has an aluminium hydroxide and oil emulsion adjuvant, and Ingelvac^®^MycoFLEX has a Carbomer adjuvant. Both vaccines were used according to their Summary of Product Characteristics (SPC’s).

### Experimental design

Pigs were included in the trial just after weaning at four weeks of age and were given ear tags with an individual number in the same colour. The pigs were randomly assigned to one of three treatment groups according to a randomisation list prepared beforehand. Pigs in group 1 were injected intramuscularly (im) in the neck once with 2 mL of ThoroVAX^®^VET. Pigs in group 2 were injected im once with 1 mL of Ingelvac^®^MycoFLEX. Pigs in group 3 were included as non-vaccinated controls. The vaccination was performed by a person who was not involved in other parts of the trial and vaccination status was thereby blinded to the evaluators. The pigs from all three groups were commingled within the pens. All the pigs within an age group were included, with 1/3 left non-vaccinated and 2/3 vaccinated against *M. hyopneumoniae*.

In herd A, pigs were included at two different times (two batches), and in herd B pigs were included at three different times (three batches).

The study was approved by the Danish Health and Medicines Authority (http://www.sundhedsstyrelsen.dk; J.no. 2011081993).

### Data recordings

#### ***Clinical observations***

The pigs were observed daily for reactions to the vaccination, for clinical signs of disease and abnormalities.

#### ***Growth rate***

In order to assess ADG, all pigs were weighed individually at three different times: on the day of inclusion in the trial, when the batch was moved from the nursery to the grower/finisher unit and when the first pig in the batch was ready for slaughter.

#### ***Antibiotic use***

Individual antibiotic treatment of the pigs included in the trial was recorded daily by the farmer, who also recorded whether or not the treatment was due to respiratory symptoms. Antibiotic treatment frequency was calculated for nursery and grower/finisher pigs in each treatment group.

#### ***Mortality***

All pigs that died during the study were recorded.

#### ***Pathology***

A subset of dead pigs was necropsied to validate the cause of death at the Laboratory for Swine Diseases, Kjellerup, Denmark. A gross pathological examination was performed.

**Figure 1:**
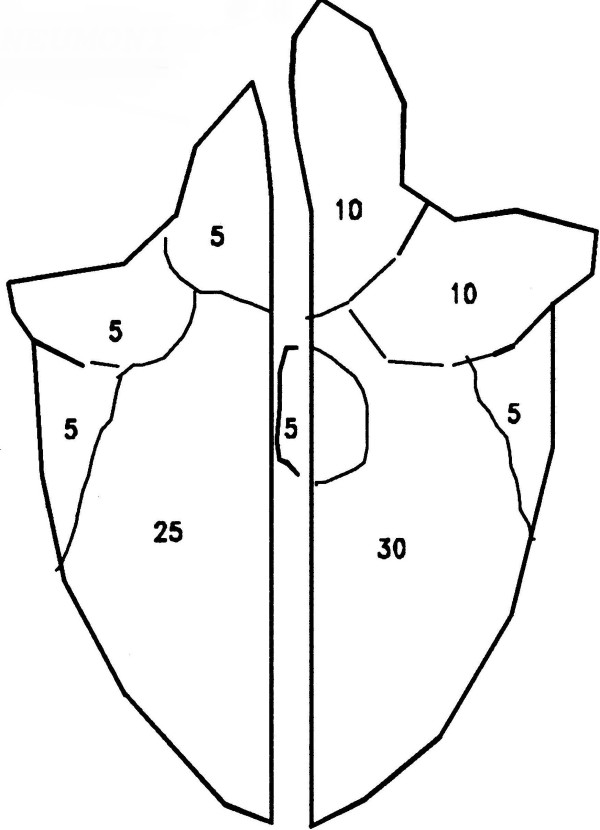
Example of how the individual lung lobs contributed to the calculation of the total lung volume.

Pigs were slaughtered when they reached market weight (110 kg). At the abattoir, the lung from each pig was collected and marked with the treatment group number. At the Laboratory for Swine Diseases, Kjellerup, Denmark the lung lesions were evaluated as described by Christensen *et al.*[2]. Briefly, each lung was scored yes/no with regard to presence of uncomplicated catarrhal bronchopneumonia (presumable caused only by *M. hyopneumoniae*), complicated catarrhal bronchopneumonia (presumable aggravated with secondary infections) or fissures (appears in lobes previously affected with catarrhal bronchopneumonia). From this, the number of pigs with at least one *M. hyopneumoniae*-like lesion (lungs with uncomplicated catarrhal bronchopneumonia, complicated catarrhal bronchopneumonia or fissures) was calculated. Besides this, the volume percentage of lung tissue affected by uncomplicated or complicated catarrhal bronchopneumonia was roughly estimated [See Figure 1 for examples].

### Statistical analysis

The sample size was based on lung lesions and ADG. With regard to lung lesions, expressed as a percentage of lungs with no lesions at all, it was assumed that a difference of 13 percentage points between groups in the range of 40% - 60% could be found. With a level of confidence of 95% and a power of 80%, the sample size was 206 animals per vaccination group per herd. With regard to ADG, it was assumed that a difference of 30 g per day between groups in the range of ADG of 900 g per day and an assumed variation of 120 g per day could be found. With a level of confidence of 95% and a power of 80%, the sample size was 253 animals per group per herd.

To allow both parameters to be evaluated in the trial, it was necessary to select 250 test animals per group per herd. In order to allow for mortality, lost ear tags and missing pigs, the calculated number were increased by 20% based on previous experiences. Thus, the sample size was 300 pigs per treatment group in each herd.

The prevalence of lungs with 1) uncomplicated catarrhal bronchopneumonia, 2) complicated catarrhal bronchopneumonia, 3) fissures and 4) at least one *M. hyopneumoniae*-like lesion for each treatment group and each herd was calculated. Four separate logistic regression analyses were performed for each of the four lung lesion prevalences. Mortality was also analysed by a logistic regression.

The percentage of the total lung volume with uncomplicated catarrhal bronchopneumonia and complicated catarrhal bronchopneumonia lesions, ADG and antibiotic treatment frequency were used as an outcome in three separate linear regressions. ADG and antibiotic treatment were compared for nursery and grower/finisher pigs separately. The start weight was included as a fixed effect in models with ADG.

PROC GENMOD from the statistical package SAS^®^ was used for logistic regression, and PROC MIXED was used for linear regression (SAS Inst. Inc., Cary, NC). Herd, treatment group and gender were included as fixed effects in all models. To test for different results in the herds, an interaction with herd and treatment group was included. A significance level of 5% was used.

## Results

Although only 900 pigs per herd were needed, a total of 2,256 pigs were included in the field trial. The distribution between herd and treatment group can be seen in Table 1.

**Table 1:** **Distribution of pigs in a field trial of vaccines against *M. hyopneumoniae***

**Herd**	**A**			**B**		
**Treatment group**	**ThoroVAX^®^VET**	**Ingelvac^®^MycoFLEX**	**Control**	**ThoroVAX^®^VET**	**Ingelvac^®^MycoFLEX**	**Control**
Number of pigs	379	377	393	368	363	376
Total number of pigs	1149			1107		

In herd B, one pig (vaccinated with Ingelvac^®^MycoFLEX) was missing at the end of the nursery period. At weighing before slaughter, two pigs (vaccinated with Ingelvac^®^MycoFLEX ) from herd A and 11 pigs ( four control, two vaccinated with ThoroVAX^®^VET and five vaccinated with Ingelvac^®^MycoFLEX group) from herd B were missing. A total of only 14 pigs out of 2,256 were missing before the final weighing in the two herds.

### Clinical observations

No adverse reactions were observed in any of the pigs following vaccination.

### Growth rate

In this trial, the mean weights at inclusion were equal in the three treatment groups in both herds (Table 2). No statistically significant differences for ADG were shown between the vaccinated groups and the control groups in the nursery period (*P* = 0.29) and grower/finisher period (*P* = 0.84) (Table 2).

**Table 2:** The effect of vaccinations on growth performance, antibiotic treatment and mortality

**Herd**	**A**			**B**		
**Treatment group**	**ThoroVAX^®^VET**	**Ingelvac^®^MycoFLEX**	**Control**	**ThoroVAX^®^VET**	**Ingelvac^®^MycoFLEX**	**Control**
*Nursery pigs*						
Mean weight start nursery (sd^*^)	7.2 (1.3)	7.3 (1.2)	7.2 (1.2)	6.5 (1.8)	6.5 (1.8)	6.5 (1.6)
Mean weight end nursery (sd)	32.7 (7.5)	32.8 (7.6)	32.5 (7.4)	31.6 (6.1)	32.4 (5.9)	31.4 (5.4)
ADG^**^ nursery (sd)	508 (9)	516 (9)	507 (9)	538 (10)	545 (10)	537 (10)
Pigs individually treated with antibiotics (%)	1.9	3.4	1.0	3.3	1.4	3.2
Mortality (%)	2.9	3.2	3.1	1.9	1.9	1.3
*Grower/finishers*						
Mean weight when first pig in a batch was ready for slaughter (sd)	79.4 (14.6)	79.4 (14.4)	79.1 (15.3)	76.3 (11.6)	76.9 (11.4)	75.5 (11.8)
ADG grower/finisher pigs (sd)	868 (11)	863 (11)	867 (11)	807 (11)	803 (11)	807 (11)
Pigs individually treated with antibiotics (%)	1.9	0.8	2.0	5.2	5.5	6.1
Mortality (%)	3.5	3.6	3.2	3.6	2.3	2.7

^*^Sd: Standard deviation of the mean. ^**^ADG: Average daily gain.

### Antibiotic use

In herd A, all pigs were batch medicated to avoid oedema disease for the first three weeks after entry into the nursery unit. Besides this, no batch medication was used in the two herds. A few pigs were treated for respiratory symptoms. In herd B, three nursery pigs (one control pig and two ThoroVAX^®^VET-vaccinated pigs) and six grower/finisher pigs (three ThoroVAX^®^VET-vaccinated pigs and three Ingelvac^®^MycoFLEX-vaccinated pigs) were treated. In herd A, no individual treatment was given due to respiratory symptoms.

In herd A, individual treatment was given to 1.0% - 3.4% of the nursery pigs. In herd B, 1.4% - 3.3% of the nursery pigs were treated with antibiotics. In the grower/finisher period, 0.8% - 2.0% of the pigs in herd A were treated with antibiotics, and in herd B 5.2% - 6.1% of the pigs were treated (Table 2). There were no statistically significant differences in the percentage of pigs treated with antibiotics for nursery pigs (*P* = 0.54) and grower/finisher pigs (*P* = 0.38) in the two herds.

### Mortality

The mortality for nursery pigs in herd A was between 2.9% and 3.2% and in herd B between 1.3% and 1.9% in the treatment groups (Table 2). The higher mortality in herd A was mainly due to oedema disease. For grower/finishers, the mortality in herd A was between 3.2% and 3.6% and for herd B between 2.3% and 3.6% in the treatment groups (Table 2). No statistically significant differences between the treatment groups were observed for nursery pigs (*P* = 0.90) or grower/finishers (*P* = 0.73).

From herd A, 13 pigs (six control, four vaccinated with ThoroVAX^®^VET and three vaccinated with Ingelvac^®^MycoFLEX) were necropsied, and for herd B six pigs (two control, three vaccinated with ThoroVAX^®^VET and one vaccinated with Ingelvac^®^MycoFLEX) were necropsied. The unequal distribution of necropsied pigs was due to the blinding, which made it impossible to know which treatment the pig had received. None of the necropsied pigs (n = 19) died due to respiratory diseases.

### Pathological findings

A total of 1,831 lungs were collected at the abattoir (867 from herd A and 964 from herd B). A further 176 lungs from herd A and 65 lungs from herd B were lost due to technical reasons, with an equal distribution between the three treatment groups for these missing lungs.

**Figure 2:**
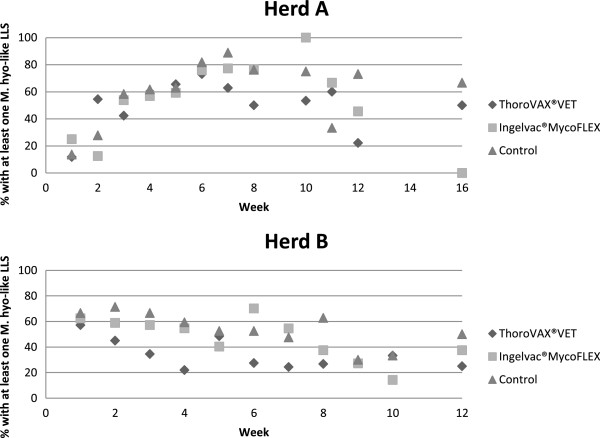
**Prevalence of pigs with at least one *Mycoplasma hyopneumoniae*-like lung lesion in each treatment group, at each delivery date.**

The prevalence of pigs with at least one *M. hyopneumoniae*-like lung lesion in each treatment group, at each delivery date, can be seen in Figure 2. Fluctuations in the prevalence of pigs with at least one *M. hyopneumoniae*-like lung lesion were seen over time.

The interaction between herd and treatment group was statistically significant in the models with uncomplicated catarrhal bronchopneumonia, number of lungs with at least one *M. hyopneumoniae*-like lesion and fissures due to different levels of lung lesions within the herds. Therefore, the results from these models are reported for each herd individually.

Statistically significantly fewer pigs vaccinated with ThoroVAX^®^Vet had uncomplicated catarrhal bronchopneumonia compared with the control group of non-vaccinated pigs (Herd A: *P* =0.05; Herd B: *P* < 0.0001). There were no statistically significant differences between pigs vaccinated with Ingelvac^®^MycoFLEX compared with the control group of non-vaccinated pigs for uncomplicated catarrhal bronchopneumonia (Herd A: *P* = 0.97; Herd B: *P* = 0.29) (Table 3).

**Table 3:** The prevalence of lung lesions and mean percentage of the total lung volume with uncomplicated and complicated catarrhal pneumonia lesions (volume %) at slaughter

**Herd**	**A**			**B**		
**Treatment group**	**ThoroVAX^®^VET**	**Ingelvac^®^MycoFLEX**	**Control**	**ThoroVAX^®^VET**	**Ingelvac^®^MycoFLEX**	**Control**
N^*^ lungs obtained	285	283	299	324	306	334
N uncomplicated catarrhal pneumonia (%)	130^b^	152^a^	161^a**^	51^b^	104^a^	127^a^ (38.0)
	(45.6)	(53.7)	^*^(53.9)	(15.7)	(34.0)	
N complicated catarrhal pneumonia (%)	0	2	4	1	8	1
	(0)	(0.7)	(1.3)	(0.3)	(2.6)	(0.3)
N fissures (%)	53^a^ (18.6)	53^a^ (18.7)	50^a^ (16.7)	63^b^ (19.4)	86^a^ (28.1)	104^a^ (31.1)
N of pigs with at least one *M. hyopneumoniae* like lung lesion (%)	156^b^ (54.7)	171^a^ (60.4)	189^a^ (63.2)	105^b^ (32.4)	159^a^ ( 52.0)	185^a^ (55.4)
Volume (%) (sd)^***^	9.0^b^	10.7^a^	11.9^a^	6.3^b^	8.6^a^	9.0^a^
	(7.1)	(8.4)	(9.1)	(4.4)	(6.7)	(7.6)

^*^N: Number. ^**^a/b indicated significant (*P* <0.05) difference from the reference group “Control”. ^***^Sd: standard deviation of the mean.

The prevalence of pigs with complicated catarrhal bronchopneumonia was very low, and therefore no statistical analysis was performed for complicated catarrhal bronchopneumonia (Table 3).

Statistically significantly fewer pigs vaccinated with ThoroVAX^®^Vet had at least one *M. hyopneumoniae*-like lung lesion compared with the control group of non-vaccinated pigs (Herd A: *P* = 0.04; Herd B: *P* < 0.0001 ). For Ingelvac^®^MycoFLEX, no statistically significant differences were found when comparing to the control group (Herd A: *P* = 0.49; Herd B: *P* = 0.39) (Table 3).

For fissures, a statistically significant difference was found when comparing pigs vaccinated with ThoroVAX^®^Vet with the control group in herd B (*P* = 0.0006) but not in herd A (*P* = 0.55). When comparing the Ingelvac^®^MycoFLEX-vaccinated pigs with the control group, no statistically significant differences were found (Herd A: *P* = 0.52; Herd B: *P* = 0.40).

The percentage of the total lung volume with uncomplicated and complicated catarrhal bronchopneumonia was statistically significantly lower for ThoroVAX^®^Vet-vaccinated pigs compared with the control group of non-vaccinated pigs (*P* = 0.001). There were no statistically significant differences between pigs vaccinated with Ingelvac^®^MycoFLEX compared with the control group of non-vaccinated pigs when comparing the percentage of the total lung volume with uncomplicated and complicated catarrhal bronchopneumonia (*P* = 0.16) (Table 3).

## Discussion

The prevalence of lung lesions related to *M. hyopneumoniae* was statistically significantly reduced for pigs vaccinated with ThoroVAX^®^VET but not for pigs vaccinated with Ingelvac^®^MycoFLEX compared with a non-vaccinated control group in two modern Danish pig production sites with a high level of biosecurity. There was no statistically significant effect of vaccination on growth rate, antibiotic consumption and mortality. It was surprising that no difference was found between lung lesions and ADG, since this correlation has been observed in several studies e.g. [8-10], although missing in another [11].The lack of correlation between lung lesions and growth rate, together with the low ADG seen in the study, could be explained by the fact that a batch of pigs was weighed when the first pig within the batch reached slaughter weight. At that time, the average weight was 75–80 kg, with a normal slaughter weight of 100–110 kg, and therefore some pigs had to remain in the grower/finishing unit for a further seven to eight weeks before being sent for slaughter. Furthermore, it was decided not to put restrictions on the normal delivery strategy for the herds, forcing the farmer to deliver pigs before they had reached slaughter weight. If the infection with *M. hyopneumoniae* was late, which the large number of pigs with catarrhal bronchopneumonia indicates, the ADG would be most reduced late in the grower/finishing phase. In this phase, pigs vaccinated with ThoroVAX^®^VET seemed to be best protected, since they had the lowest prevalence of lung lesions. Therefore, it could be hypothesised that the ThoroVAX^®^VET-vaccinated pigs would have a higher ADG, compared with the control, if all of the pigs had been weighed just before slaughter.

Overall, no benefit of vaccination was found in antibiotic treatment. A few vaccinated pigs were treated for respiratory diseases. The daily treatment was given by the farmer, and, despite careful training of the farmer in clinical observation, it could be questioned whether the observations were made correctly.

This trial was conducted in modern facilities with high performance and a good level of biosecurity. Nevertheless, ThoroVAX^®^VET was found to reduce the prevalence of lung lesions related to *M. hyopneumoniae* significantly. The best effect of vaccination was found in Herd B, although a good effect was also found in herd A. It is important to note that there were fluctuations in the prevalence of lung lesions related to *M. hyopneumoniae* over time for both herds. At one point, even the control non-vaccinated pigs had the lowest prevalence of lung lesions related to *M. hyopneumoniae*. The diverging effects in efficacy between herds and over time emphasize the complexity of the porcine respiratory disease complex. Therefore, it is important not only to make one evaluation of lung lesions when investigating the reason for a respiratory problem within a herd. It should always be supplemented with detection of the agent/agents and/or blood samples etc.

The effect of both vaccines might be underestimated in this study, since only parts of the total populations were vaccinated. This might lead to a lower level of protection at herd level, and, if non-immune pigs develop disease, they will increase the general pathogen load - not only associated with *M. hyopneumoniae* but the porcine respiratory disease complex in general. Therefore, vaccination against *M. hyopneumoniae* might still be an economic benefit in modern pig production with a high level of biosecurity despite the lack of increased ADG in this study.

## Conclusion

The prevalence of lung lesions related to *M. hyopneumoniae* were statistically significantly reduced for pigs vaccinated with ThoroVAX^®^VET but not for pigs vaccinated with Ingelvac^®^MycoFLEX^®^ compared with a non-vaccinated control group.

## Abbreviations

SPF: Specific pathogen free; i.e.: free of infection with *Mycoplasma hyopneumonia*, *Actinobacillus pleuropneumoniae* (serotypes: 1, 2, 3, 4, 5, 6, 7, 8, 9, 10, 12) Toxigenic *Pasteurella Multocida, Brachyspira hyodysenteriae*, Porcine Reproductive and Respiratory Syndrome virus (type 1 and 2), Sarcoptes scabiei var. suis and Haematopinus suis.

## Competing interests

The study was financed and monitored by MSD Animal Health but was conducted solely by the Pig Research Centre.

The authors have no financial or personal relationship with people or organisations that could inappropriately influence or bias the content of this paper.

## Authors’ contributions

CSK designed the study, selected the herds, assisted with farm visits and data collection, assisted with the statistical analysis and drafted the manuscript. JV performed the statistical analysis. BS carried out the pathological investigations. PB participated in designing the study and critically revised the manuscript. All authors read and approved the final manuscript.
